# Regulatory Effect of Melatonin on Cytokine Disturbances in the Pristane-Induced Lupus Mice

**DOI:** 10.1155/2010/951210

**Published:** 2010-07-20

**Authors:** Ling-ling Zhou, Wei Wei, Jun-feng Si, Dong-ping Yuan

**Affiliations:** ^1^Key Laboratory of Anti-inflammatory and immunopharmacology in Education Ministry of China, Institute of Clinical Pharmacology, Anhui Medical University, Hefei 230032, China; ^2^Jiangsu Provincial Key Laboratory of Pharmacology and Safety Evaluation of Material Medica, Department of pharmacology, Nanjing University of Traditional Chinese Medicine, No. 282 Hanzhong Road, Nanjing 210029, China; ^3^Institute for Biomedical Electronic Engineering, Nanjing University, Nanjing 210093, China

## Abstract

Systemic lupus erythematosus (SLE) develops in relation to many environmental factors. In our opinion, it is more important to investigate the effect of melatonin on the environmental- related SLE. In the present study, 0.5 ml pristane were used to induce SLE in female BALB/c mice. Melatonin (0.01, 0.1, 1.0 mg/kg) was orally administered immediately after pristane-injection for 24 weeks. IgM anti ssDNA and histone antibodies were detected after 0, 1, 2, 4, 8 weeks pristane injection. The levels of IL-2, IL-6 and IL-13 were detected after 24 weeks. Renal lesions were also observed. The results showed that melatonin antagonized the increasing levels of IgM anti ssDNA and histone autoantibodies. Melatonin could also decrease the IL-6 and IL-13 production and increase the IL-2 production. Besides, melatonin could lessen the renal lesions caused by pristane. These results suggested that melatonin has a beneficial effect on pristane-induced lupus through regulating the cytokines disturbances.

## 1. Introduction

 Systemic lupus erythematosus (SLE or Lupus) is an autoimmune disease that can attack the body's normal tissue and cells, resulting in inflammation and tissue damage [1, 2]. SLE occurs at any age and in any gender. However, women are more likely to have SLE than men [3, 4]. Besides, disturbance in the cytokine network has also been reported in SLE [5], including IL-1, IL-2, IL-6, IL-13, and IFN-*α*. These cytokines have close relations to the development of SLE such as autoantibodies production and immune-complex nephritis. But there are some contradictory results about the changes of some cytokines in different reports.

In recent years, more and more attention has been paid to environmental factors that may be implicated in the pathogenesis of SLE [6, 7]. Autoimmune diseases are becoming increasingly common in industrialized countries, and these diseases can be influenced by environmental factors [8]. Pristane is a likely candidate as an environmental trigger of SLE in susceptible population. Animal experiments showed that pristane could induce lupus-like autoimmune disease symptoms in a strain of mice (BALB/c), such as high levels of autoantibodies and immune-complex glomerulonephritis [9, 10]. Some epidemiological investigations also proved that all of the persons with pristane in their blood had distinct autoimmune diseases or symptoms of autoimmune disease [11]. In addition, pristane found in mineral oil, shark oil, and many foods seems to be a possible environmental exposure that may trigger SLE. So it might be more appropriate to research environmental factors involved in SLE by using pristane-induced SLE-like murine model. 

Melatonin is synthesized and secreted mainly by the pineal gland that can make specific receptors in and out of the central nervous system. Melatonin not only can directly affect inflammation and immune cells, but also has indirect influences on it through thalamencephalon [12]. More importantly, melatonin is regarded as an important active substance in the neuro-immune-endocrine system [13]. It is able to regulate the imbalance of cytokine network in some autoimmune diseases such as rheumatoid arthritis and adjuvant arthritis [14, 15]. Some experiments investigated its effects on MRL-lpr/lpr mice and showed that melatonin was beneficial for spontaneous SLE in female mice [16]. Since environment is an important factor in SLE in modern society, we were more interested in the effects of melatonin on the environmental-related SLE. 

To investigate the role of melatonin in SLE, especially in the environmental-related lupus, pristane-induced mice were used. The effects of melatonin on the cytokine disturbances and the following changes in pristane-induced SLE model were also determined.

## 2. Materials and Methods

### 2.1. Animals

 Sixty female BALB/c mice aged two months (17 ± 2 g) were supplied by the Experimental Animal Center of Anhui Medical University. All experimental protocols described in this study were approved by the Ethics Review Committee for Animal Experimentation of Institute of Clinical Pharmacology, Anhui Medical University.

### 2.2. Materials

 Melatonin was purchased from Sigma. Pristane, heat-denatured calf thymus DNA (ssDNA), total calf thymus histone (histone), concanavalin A (ConA), and lipopolysaccharides (LPS) were also from Sigma. Biotin-conjugated rabbit-anti-mouse IgM antibodies and horseradish peroxidase-labeled avidin were purchased from S_ABC_. Mouse interleukin-2 ELISA kits and interleukin-6 ELISA kits were purchased from ADL. Mouse interleukin-13 ELISA kits were purchased from BIOO.

### 2.3. Experimental Protocols

 Sixty female BALB/c mice were randomly divided into six groups: normal control group, model group, prednisone-treatment group which served as positive control group, and melatonin (0.01, 0.1, 1.0 mg/kg) treatment groups including melatonin group one (MT1), melatonin group two (MT2), and melatonin group three (MT3). The mice in normal control group were given an intraperitoneal injection of 0.5 ml normal saline (NS), and the other groups were given an intraperitoneal injection of 0.5 ml pristane. The mice in normal control group and model control group received intragastric administration of NS per day after the first treatment. The mice in positive control group were given intragastric administration of 5 mg/kg prednisone (Pre) per day. The mice in MT1 group were intragastrically treated with 0.01 mg/kg melatonin, MT2 0.1 mg/kg melatonin, and MT3 1.0 mg/kg melatonin. Sera were collected from the tail vein before treatment (0) and 2, 4, and 8 weeks after treatment to measure the level of autoantibodies. Twenty-four weeks after pristane administration, all mice were killed, and spleens were removed for immunological detections; meanwhile, nephric tissues were examined by light microscopy.

### 2.4. Enzyme-Linked Immunosorbent Assays (ELISAs) for Anti-ssDNA and Histone Antibodies

 Anti-ssDNA and histone IgM antibodies were detected by an ELISA technique similar to the one as described in [17]. 96-well ELISA plates coated with 10 mg/L ssDNA or histone antigens were incubated overnight at 4°C. On the following day, plates were washed with PBS-Tween. The plates were incubated with blocking buffer (PBS-2% bovine serum albumin) overnight at 4°C and washed with PBS-Tween. After incubated overnight at 4°C with murine serum diluted to 1 : 250, the plates were washed again and then incubated for 2 hours at 4°C with biotin-conjugated-rabbit antimouse IgM antibodies diluted to 1 : 200. Subsequently, the plates were washed and incubated for 2 hours at 4°C with horseradish peroxidase-labeled avidin in 1 : 200 dilution. The plates were then washed again, and 100 *μ*L of substrate [3, 3′, 5, 5′]—tetramethylbenzidine (TMB) and hydrogen peroxide solution were added to the wells for 30 minutes at 37°C. The reaction was terminated by addition of 50 *μ*L of 2 M H_2_SO4 per well. The intensity of the yellow color was read at 490 nm in a microplate reader. The sera of other eight normal BALB/c mice were collected and detected at the same time, and the mean density and SD were calculated to serve as control. The mean enzyme indexes (EI) of the samples in different groups were calculated as:

(1) $$\text{EI}=\frac{\text{intensity}\text{of}\text{the}\text{sample}}{\text{mean}\text{intensity}\text{of}\text{control}\text{mice}+3\text{SD}}\times 100.$$

### 2.5. Spleen Cell Culture and Induction of Cytokine

 Spleen cell Culture and induction of cytokines were modifications of the methods as described in [18, 19]. Briefly, single-cell suspensions were prepared from spleens aseptically removed from mice and cultured in 24-well culture plates at a final concentration of 1 × 10^6^ cells/ml. The cells were cultured in RPMI-1640 supplemented with 10% fetal calf serum, 2 mM glutamine, 1 mM sodium pyruvate, 50 *μ*M 2-mercaptoethanol, and 10 ml penicillin, streptomycin and antimycotic solution/L (Sigma). Cultures were stimulated with 3 mg/L ConA, 12 mg/L LPS, or medium alone and incubated at 37°C in humidified air with 5% CO_2_. The supernatant was harvested at 48 h and preserved at −20°C for cytokine assays.

### 2.6. Cytokine Assays

 The concentrations of interleukin-2 (IL-2), interleukin-6 (IL-6), and interleukin-13 (IL-13) in the supernatant were determined using appropriate commercial ELISAs for the murine form of these cytokines. The intensity of each sample was read at 450 nm in a microplate spectrophotometer.

### 2.7. Histopathological Techniques

 The kidneys collected while sacrificing mice were fixed for 24 hours in neutral buffered formalin prior to paraffin embedding. Sections were stained with Haematoxylin and Eosin and Jones silver methenamine and then examined under a light microscope for the severity of the renal lesion. Glomerulonephritis, renal tubular lesions and interstitial inflammation were observed, and the degree of severity of histological lesions characteristic of lupus nephritis was evaluated as absent (−), mild (+), moderate (++), and severe (+++).

### 2.8. Statistical Analysis

 Results were expressed as the mean ± SD. Statistical analysis was performed using Student's two-way* t*-test with *P* < .05 as the minimal level of significance

## 3. Results

### 3.1. Effects of Melatonin on Levels of IgM Anti-ssDNA and Histone Antibodies in Sera

**Figure 1:**
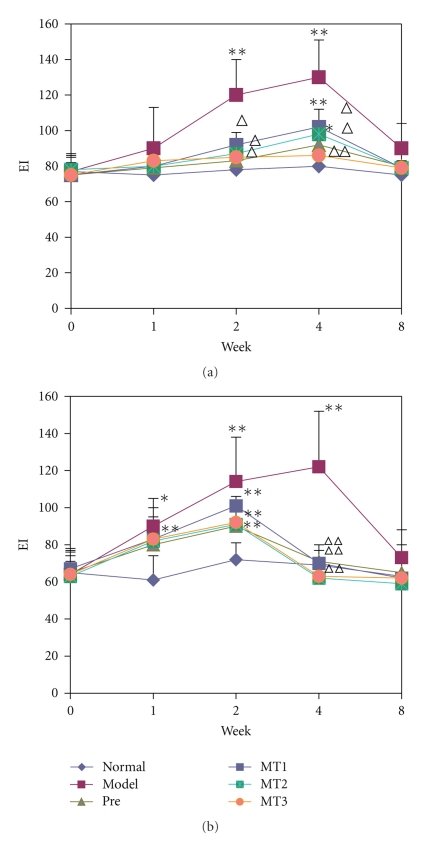
The sera of mice in each group were collected before treatment (0), 2, 4, and 8 weeks later, and levels of IgM anti-ssDNA and Antihistone antibodies were detected by ELISA. EI was calculated according to the formula. (a) the level of IgM anti-ssDNA antibody in each group, (b) the level of IgM Antihistone antibody in each group. Data were given in mean ± SD (*n* = 6–8). *P < .05, **P < .01 versus sera at 0 wk, ∆*P* < .05, ∆∆*P* < .01 versus sera in model control mice.

The levels of IgM anti-ssDNA and histone antibodies were significantly different between pristane-injection and melatonin treatment groups (*P* < .05, Figures 1(a) and 1(b)).

Two weeks after a single intraperitoneal injection of pristane, the level of anti-ssDNA IgM antibodies in sera began to increase obviously, reached peak at 4 wk, then began to decrease, and finally returned to normal at 8 wk. In MT1 group, the level of anti-ssDNA IgM antibodies in sera also increased and reached peak at 4 wk, but then decreased more obviously compared to the model control group (*P* < .05). In MT2 group, the level of anti-ssDNA IgM antibodies in sera increased during the first four weeks, but much lower than that of model group (*P* < .05), and remained normal in other periods. In MT3 group, the level of anti-ssDNA IgM antibodies did not increase (Figure 1(a)). 

Antihistone antibodies in sera increased 1 wk after injection, reached peak at 4 wk, then decreased gradually, and returned to normal at 8 wk. In MT1 group, the levels of Antihistone antibodies in the sera were significantly lower than that of the model control group (*P* < .01) and were back to normal at 4 wk. In MT2 and MT3 groups, the levels of antibodies increased during the first four weeks, but much lower than model group (*P* < .01), and remained normal in the other periods (Figure 1(b)).

### 3.2. Effects of Melatonin on Cytokines Production

**Figure 2:**
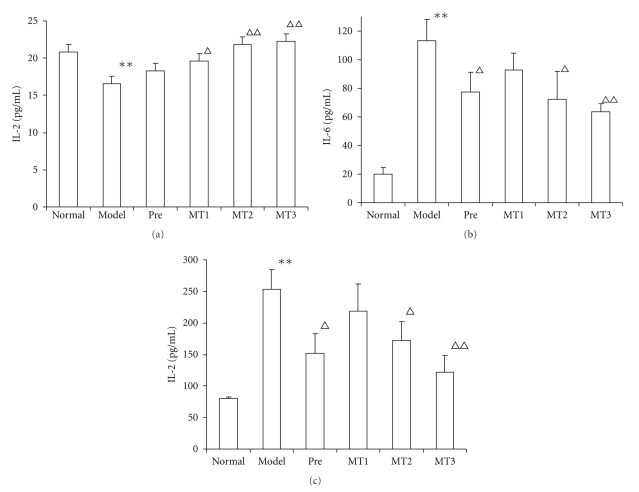
All mice were sacrificed at the end of 24 weeks, and splenic lymphocytes were seeded at 1 × 10^6^ cells/well. IL-2 concentrations in splenic lymphocytes were stimulated for 48 h with 3 mg/L ConA. IL-6, and IL-13 concentrations in splenic lymphocytes were stimulated for 48 h with 12 mg/L LPS. IL-2, IL-6, and IL-13 concentrations in culture supernatants were detected by ELISA. Data were given in mean± SD (*n* = 4–5). **P < .01, *P < .05 versus Normal control group, ∆*P* < .05, ∆∆*P* < .01 versus Model control group.

To gain a better insight into the influence of melatonin on cytokines in SLE, production of Th1-type and Th2-type cytokines by splenocytes stimulated with ConA or LPS was assayed during the course of the murine lupus. The results showed that the production of IL-2, IL-6, and IL-13 changed in pristane-induced SLE mice (Figure 2). IL-2 production of splenocytes from mice in model control group was lower than that from normal mice (*P* < .05), while IL-6 and IL-13 production of splenocytes from mice in model control group was higher than that from normal mice (*P* < .01). In MT2 and MT3 groups, IL-2 levels were up to the normal level and higher than that of model mice obviously (*P* < .05), while the IL-6 and IL-13 levels were lower than that of the model mice obviously (*P* < .05, .01) (Figure 2).

### 3.3. Effects of Melatonin on Renal Histopathological Changes

**Figure 3:**
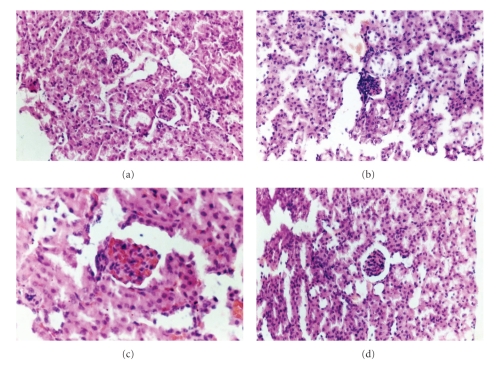
The kidneys collected at the time of sacrifice were stained with H&E for histological examination. (a) Normal control group (HE × 200). (b) Model control group (HE × 200). (c) Model control group (HE × 400). (d) MT group (HE × 200).

The renal histopathological changes of the mice are shown in Table 1 and Figure 3. 

As shown in Figure 3(a), renal abnormalities were not seen in normal mice. The volume and the cell numbers of glomeruli were normal. Glomerular capillary wall was thin. No protein cast was seen in tubule. Lymphocytes infiltration and proliferation of fibroblasts were not seen in renal interstitial.

As shown in Figures 3(b) and 3(c) and Table 1, most pristane-injected mice revealed renal abnormalities ranging from mild to severe patterns. Some glomeruli showed glomerular atrophy with capillaries dilation and thickening of capillary walls. Some cites revealed thrombus formation. Mesangial broadening and cell layers increasing were also observed. Glomerular capsule wall was thick, and the space between capsule wall and endothelial cells disappeared in severely affected glomerulus. Some renal tubules showed dilation and the epithelial cells proliferation. Protein casts were seen in tubule. An infiltration of inflammatory cells such as monocytes, lymphocytes, and plasmacytes with focal aggregation was seen in renal interstitial. Proliferations of interstitial fibroblasts were also observed.

There were some improvements of different degrees in melatonin-treated mice (Figure 3(d) and Table 1). Glomerular atrophy and thickening of capillary walls were changed to a mild or moderate degree. Mesangial broadening was also improved. The space between capsule wall and endothelial cells was smaller than that of normal mice but there was an improvement compared to the nontreated model group. Slight lymphocytes infiltration was also observed. Some renal tubules showed mild dilation and a few protein casts. Nevertheless, all the changes were less severe than that in model mice.

## 4. Discussion

In this study, the immune disturbances of pristane-primed mice and the effects of melatonin were observed. Since anti-DNA and histone antibodies were sensitive in SLE [20, 21], we investigated the changes of IgM anti-ssDNA and Antihistone antibodies and found high levels of autoantibodies in the pristane-injected mice. Renal lesions were also found in the mice. These results were consistent with the previous reports. We also found that melatonin antagonized the increasing levels of IgM anti-ssDNA and histone autoantibodies, and melatonin could lessen the renal lesions caused by pristane. Pristane is a peritoneal irritant. It is well known that IgM class are produced by the B1 (CD5+) B-cell subset [22], and the B1 subset is highly enriched in the peritoneal cavity and is expanded in BALB/c mice [23], raising the possibility that IgM anti-ssDNA and Antihistone antibodies induced by i.p. pristane are derived from the subset [24]. Previous studies reported that melatonin had effects on the first antibody immune response (IgM, IgG) [25]. Some studies indicated that melatonin had two-way modulation on lymphocytes [26]. So we thought that melatonin could improve the SLE symptoms by modulating the overproduction of B cells.

Cytokines network plays an important role in the development of SLE. In some *in vitro* experiments, melatonin could inhibit the immune effects of some cytokines and T lymphocytes [27]. So we observed the effects of melatonin on the disturbance of cytokines network. IL-6 is thought as an inflammatory cytokine which is secreted by a variety of tissue cells, including macrophages and Th2-cells. IL-6 level has positive correlation to the activity index of SLE. Our results showed that the level of IL-6 was increased after pristane injection, while melatonin could decrease the IL-6 production. The increasing level of IL-6 could cause B cells to secret more antibodies and promote the development of nephritis [28]. So the downregulation of IL-6 production by melatonin is helpful in inhibiting the antibodies production and renal lesions.

IL-2 is secreted by Th1-cells, and IL-13 is secreted by Th2-cells. There are some contradictory results in the documents about the changes of IL-2 and IL-13. So in this pristane-induced SLE model, we investigated the levels of the two cytokines. Our results revealed that the level of IL-2 in the pristane-injected mice was lower than that in the normal mice, while the level of IL-13 in the pristane-injected mice was higher than that in the normal mice. We also found that melatonin could decrease the IL-13 production but increased the IL-2 production. Many reports showed that the level of IL-2 decreased in the SLE, especially in the active stage, and that IL-2 could down-regulate the B lymphocytes activation [29]. Some researches showed that melatonin could regulate the level of IL-2/IL-2 receptors [30], which may be the mechanism of the inhibitory action of melatonin on IL-2 production. IL-13, one of the most important cytokines in the lupus, was believed to be a B-cell activating factor which resulted in the excessive production of many autoantibodies [31, 32]. IL-13 has relation to the renal lesions of SLE [33]. So the downregulation of IL-13 production by melatonin is also helpful in inhibiting the antibodies production and renal lesions. 

The decreasing level of Th1-type cytokine and the increasing levels of Th2-type cytokine also indicated that there was a shift from Th1 towards Th2 in pristine-induced lupus. Some reports showed exogenous melatonin could activate the CD_4_^+^ T cells and significantly promote the Th1-type cytokines, which participate in regulating the immune disturbances [34]. Our results showed that melatonin could upregulate the Th1-type cytokines and downregulate the Th2-type cytokines. And that indicated melatonin could modulate the disturbance of cytokine network in the SLE mice by regulating the imbalance of Th1/Th2.

Prednisone, which is a common drug for SLE [35], was served as the positive drug in this study. We found that prednisone had a regulatory effect on pristane-induced SLE. Compared to prednisone, melatonin had a similar or a little prior effect on the SLE symptoms. As we have known, the adverse reactions of prednisone are more common and serious than melatonin. Thus we thought melatonin may be a potential drug for SLE.

In conclusion, pristane-induced lupus displays disturbance in immune systems, such as high level of autoantibodies and imbalance of cytokine network, which induces the followed nephritis. The changes are similar to the human lupus, and pristane is common in the environment; thus this model is appropriate to study the effects of melatonin on environmental-related SLE. On the other hand, we also demonstrated that the administration of melatonin inhibited the changes in the pristane-induced lupus. And that suggested melatonin had a beneficial effect on environmental-related lupus. The mechanisms might involve its modulation of disturbances in the immune system, especially in cytokines network. However, further studies should be necessary for a better understanding of the regulating process and the key factor for its action on autoimmune diseases.

## Figures and Tables

**Table 1:** Renal histopathological features of the mice in different groups (*n* = 6–8). The degree of severity of histological lesions was evaluated as absent (−), mild (+), moderate (++), and severe (+++).

Group	Glomerulonephritis			Renal tubular lesions			Interstitial inflammation	
	Atrophy	Capillary walls thickening	Mesangial broadening	Dilation	Epithelial cells proliferation	Protein casts	Inflammatory cells infiltration	Fibroblasts proliferation
Normal	−	−	−	−	−	−	−	−
Model	++	++	++/+++	++	++/+++	++	++/+++	++
Pre	+	+	+	+/++	+/++	− ∼ ++	+	−
MT1	++	++	++	++	++	+	++	++
MT2	++	+	+	+	+	− ∼ ++	+	−
MT3	+	−/+	+	−/+	−/+	−/+	+	−
